# 

**Published:** 1909-06-15

**Authors:** 


### ANNOUNCEMENTS.

THE CALIFORNIA STATE DENTAL ASSOCIATION and the Alumni Association College of Dentistry, University of California, have secured the services of Dr. John Q. Byram, of Indianapolis, and Dr. Weston A. Price, of Cleveland, for their Joint Session to be held on July 6th, 7th and 8th, at the College of Dentistry, San Francisco. The presence of these men is a guarantee of a first class meeting.

MICHIGAN STATE DENTAL SOCIETY. This society will meet June 29th to July 1st, inclusive, in Kalamazoo. Unusual efforts are planned to make this one of the greatest summer meetings, and a good program is already well in hand. The papers are all to be of the technical order and the formal discussion will be written and therefore well considered. A large number of excellent clinics are assured and a manufacturer's exhibit will be held in connection with the meeting. Every ethical practitioner is cordially invited and will be welcomed.

---

INDIANA STATE DENTAL SOCIETY. This society will meet June 29th to July 1st at Indianapolis. An excellent program is in preparation and a profitable meeting is assured to all who attend.

---

TEXT BOOK ON DENTAL MATERIA MEDICA AND THERAPEUTICS. Dr. Herman Prinz announces for an early date the publication of a work on this subject. It will be a work of 650 pages. Everyone who knows Dr. Prinz will appreciate the value of such a book from his pen. He is an original worker of long experience in this line and undoubtedly will produce a work on this subject that will command respect and confidence. The profession needs a good book on Materia Medica and Therapeutics as all we now have are out of date.

---

THE DENTAL INDEX BUREAU. At a meeting of the National Institute of Dental Pedagogics, held in St. Louis last December, a committee was appointed to establish a Dental Index Bureau. Briefly stated, it is intended that this committee shall employ a competent person to review and classify all or the articles in ten or twelve of our leading dental journals, and shall furnish to each of the subscribers to the

Bureau, at frequent intervals, cards so classified and grouped that the subscriber may easily and quickly find all the articles on any subject in dentistry. These cards will be made on a definite system, by which additional cards may be added as they are received and always go to their proper places; so that no matter how many additions may be made from year to year, all of the cards on each subject will be in a separate group. For example, all of the articles on the subject of filling teeth with gold will be in one group, all on filling teeth with amalgam in another, all on cavity preparation in another, all on alveolar abcess in another, etc.

It is hardly necessary to mention the value of such an index to the wide-awake members of the profession. Journals which may have accumulated, and which are now almost worthless on account of the difficulty in finding articles when desired, will become of service as the index is extended to cover them. The man who has occasion to look up a particular subject, or to write a paper, can find all that has been written on that subject instantly. The grouping of articles on each subject enables one to bring together the views of many writers. Post-graduate courses of study can be instituted by dental societies or undertaken by individuals; the ready reference index will make college libraries of the greatest value to the faculty and to the student body; editors and writers will find their work facilitated, and the result of their study more gratifying to themselves and more highly appreciated by their readers.

The idea of the committee is to organize a permanent society for the purpose of classifying and indexing all dental literature. The present plan is but preliminary to the great work, which will cost less and less to individual subscribers as the number of members is increased. Those who help to start the work will profit more than those who follow, and will have the satisfaction of participating in one of the greatest advance movements in dentistry.

The subject-matter of several of the leading dental journals has already been classified and indexed by Dr. Arthur

D. Black, at a great expenditure of time and money. This index contains about 40,000 cards, cataloging articles in various journals from 1839 to date; but it is not available for obvious reasons, to the profession at large. Your committee is privileged to copy these cards, should the funds subscribed permit, which would be of great benefit to the subscribers and incidentally to the whole profession.

It is the committee's intention to this year furnish each subscriber with author and subject cards for all original articles, papers read before societies, book reviews, and editorials, in about ten journals for 1908 and 1909,—from 4,000 to 5,000 cards, all properly arranged with all cards for each subject together, with guide cards for each subject, and with author cards arranged alphabetically. It is expected that in 1910 the cards will be furnished for the same journals for the years 1907 and 1910; and in 1911 cards for 1906 and 1911. Thus in three years each subscriber will receive cards for these journals for six years—probably 25,000 to 30,000 cards. These cards will be sent in boxes in which they may be kept permanently. The number of journals indexed and the number of cards sent out by the committee, however, will depend upon the number of subscribers.

The preliminary report of this committee, which was made at the St. Louis meeting, gives an outline of the plan. It will be noticed that it is the intention to try this plan out for a period of three years, and subscriptions at the rate of Twenty-five Dollars ($25,00) a year for that period may be sent to any member of the committee.

Subscriptions will be received from individuals, dental Journals, dental schools, or dental societies. Any person or group of persons may send in a single subscription, and one set of cards will be sent in return. Subscription blanks may be had from any member of the committee. For full particulars write to Dr. W. L. Fickes, 6200 Penn. Ave., Pittsburg, Pa.

